# ‘HypothesisFinder:’ A Strategy for the Detection of Speculative Statements in Scientific Text

**DOI:** 10.1371/journal.pcbi.1003117

**Published:** 2013-07-25

**Authors:** Ashutosh Malhotra, Erfan Younesi, Harsha Gurulingappa, Martin Hofmann-Apitius

**Affiliations:** 1 Department of Bioinformatics, Fraunhofer Institute for Algorithms and Scientific Computing, Sankt Augustin, Germany; 2 Rheinische Friedrich-Wilhelms-Universität Bonn, Bonn-Aachen International Center for Information Technology, Bonn, Germany; 3 Molecular Connections Pvt. Ltd., Basavanagudi, Bangalore, India; University of Chicago, United States of America

## Abstract

Speculative statements communicating experimental findings are frequently found in scientific articles, and their purpose is to provide an impetus for further investigations into the given topic. Automated recognition of speculative statements in scientific text has gained interest in recent years as systematic analysis of such statements could transform speculative thoughts into testable hypotheses. We describe here a pattern matching approach for the detection of speculative statements in scientific text that uses a dictionary of speculative patterns to classify sentences as hypothetical. To demonstrate the practical utility of our approach, we applied it to the domain of Alzheimer's disease and showed that our automated approach captures a wide spectrum of scientific speculations on Alzheimer's disease. Subsequent exploration of derived hypothetical knowledge leads to generation of a coherent overview on emerging knowledge niches, and can thus provide added value to ongoing research activities.

## Author Summary

Published speculations about possible molecular mechanisms underlying normal and diseased biological processes provide valuable input for the generation of new scientific hypotheses. However, a systematic gathering of all scientific speculation that exists in a given context is a non-trivial task and, if done manually, is laborious and time-consuming. The “HypothesisFinder” approach outlined here provides a possible solution for making scientific speculation gathering more tractable. Using a dictionary of speculative patterns, HypothesisFinder detects, collates and analyzes published speculative statements for a specific context. This can be extremely useful, particularly in reference to complex and poorly understood diseases like Alzheimer's disease. For example, by formulating a series of reasonable speculations on causes and effects, we could gain new insights into the directions of Alzheimer's disease etiology and progression. An effective literature search with the help of HypothesisFinder can support the process of knowledge discovery and hypothesis generation, which has the potential to add value to ongoing research activities.

## Introduction

Biomedical information published in the scientific articles can be grossly divided into “established knowledge” and “emerging knowledge”. Established knowledge, supported by facts and repeated, independent validation of experimental findings, is widely accepted in the scientific community; this knowledge is extensively used to understand various aspects of a biological phenomenon. In contrast, emerging knowledge refers to new and less “solid” knowledge, which represents novel findings or new thoughts. Although we routinely make extensive use of established knowledge, most of us do not utilize emerging information in a systematic fashion. Systematic analysis of emerging information could, however, help to transform speculative thoughts into testable hypotheses. In the scientific literature, particularly in articles of experimental nature, speculative statements (hedges) are frequently found [1] because expressing hypothesis based on experimental results is an important part of this sort of publications [2]. Particularly, speculative statements characterize hypotheses when linked to molecular entities (genes/proteins) and backed up by experimental evidence. Such statements are often given in hopes of stimulating further research into a topic. As an example, the following speculative sentence may represent a potential hypothesis:

“*Our findings support the notion that CSF tau and Abeta(1-42)* ***may be*** *useful biomarkers in the early identification of AD in MCI subjects*” (PMID: 14699432)

The recognition of such speculative statements in scientific text has gained interest in the recent years [3]–[6]. Several groups have investigated the use of pattern matching [3], [6] or machine learning (ML) approaches [5], [7], [8], [9] to build models for recognition of speculation in text. Both of these approaches had been shown to perform competitively when it comes to detecting speculative statements in scientific text [3]–[9]. However, there are scenarios where pattern matching approaches perform better than ML (e.g. SVM-based bag of words feature) approaches [3], [6]. Additionally, machine learning techniques require comprehensive training data sets and thorough optimization. Taking into consideration the successful strategies applied in previously conducted studies, this work reports the development of a pattern matching approach for the automatic detection of speculative patterns in text (named “HypothesisFinder”) that captures most of the existing hypothetical knowledge, independent of the indication area.

In order to demonstrate the applicability of our approach, we apply HypothesisFinder to the domain of Alzheimer's disease (AD) to demonstrate its usability and to assess its performance. Particularly in case of complex and mostly idiopathic diseases like AD where the etiology of the disease is still unclear, neuroscientists are frequently introducing working hypotheses or speculations in the following form:

*“Early cognitive deficit characteristic of early Alzheimer's disease seems to be produced by the soluble forms of beta-Amyloid protein.”*

or

*“These findings suggest that there may be apolipoprotein E (apoE) isoform-specific differences of tau regulation in AD.”*

A systematic analysis of all speculative statements on a particular topic such as molecular etiology of AD, as shown in these examples, should enable us to capture the diversity of hypotheses that exist within the literature.. Motivated by the same idea, AlzSWAN [10], one of the most comprehensive community-driven knowledge bases on AD-related information, has produced a special section called ‘SWAN Hypotheses Browser’ where manually curated hypotheses inferred from the scientific literature are made available to the user community. AlzSWAN is one of the most dynamic and most relevant scientific resources representing hypothetical knowledge on AD. However, the rapid growth of publications on AD poses a challenge to curators [11] as manual information extraction is time and resource consuming. Thus, the continued growth of such knowledge bases is confined to the pace of manual curation.

The aim of the work presented here was to build an environment with the ability to cross-link speculative statements and clinical/biomedical features. The core of the developed workflow encompasses hand-crafted knowledge-based textual patterns for detecting speculative statements in biomedical free text. Two different methods were taken for the detection of speculative statements: a pattern-based approach and a machine learning (ML)-based approach. The performance of both methods was systematically compared against each other. Furthermore, the ability of HypothesisFinder to identify speculative statements in scientific text was evaluated using a dedicated corpus (Bioscope corpus [12]) and its ability to detect statements supporting AD-related hypotheses was benchmarked against the human-curated AlzSWAN knowledge base.

## Methods

### Corpus characteristics and annotations

In order to assemble a corpus suitable for pattern development, all fields of Medline articles were searched with a text query ‘Alzheimer disease AND hypothesis’ using the PubMed search engine that retrieved 3007 abstracts as on 26-7-2012. From this initial collection of retrieved abstracts, a preliminary corpus containing 150 randomly chosen abstracts was generated (referred to as HYPO-DEV-1). All abstracts in HYPO-DEV-1 were annotated with one class named *Speculative pattern (see next section for annotation examples)*. This corpus was manually annotated for the presence of speculative text patterns that characterize scientific hypotheses. In parallel, annotation guidelines for the annotation of speculative statements were developed. First, an annotator (referred to as the *principal annotator*) participated in the annotation and guideline development process. The corpus was iteratively annotated by him along with the standardization of the annotation rules. Textual patterns annotated in the HYPO-DEV-1 were collected to form an initial version of a dictionary containing speculative patterns.

In the next step, a secondary corpus containing 200 randomly chosen, but non-overlapping, abstracts were generated (referred to as HYPO-DEV- 2). HYPO-DEV- 2 was annotated by two different annotators (referred to as the *junior ann*otators). These annotators individually annotated HYPO-DEV-2 corpus based on the previously developed annotation guidelines and their annotations were used to calculate the inter-annotator agreement (IAA). The IAA determines the quality and acceptability of the notion of ‘hypothesis or speculation’ among annotators and provides a rationale for measuring the quality of prior developed annotation guidelines. Novel patterns annotated in the HYPO-DEV-2 were used to enrich the initial version of the dictionary (i.e. based on patterns collected from the HYPO-DEV- 1 corpus).

The IAA kappa between the two junior annotators was calculated as high as 0.81, which indicates an acceptable agreement, given the complicated nature of the annotated patterns. Similar to the developmental corpus, a test corpus (referred to as HYPO-TEST) was generated by searching PubMed using the keyword ‘Alzheimer’ and then randomly selecting 200 abstracts from the 58922 retrieved abstracts (as of 26-7-2012). Two new ‘independent annotators’ used the previously developed annotation guidelines for annotating the HYPO-TEST corpus. In case of any possible conflicts occurring between the annotators concerning ‘non-overlapping patterns’, the principle annotator was involved in resolving them. The following guidelines were applied for resolving the conflicting annotations between the independent annotators.

1. No entirely new annotations were added by the principle annotator. This means that if they were not marked earlier by either of the annotators, the principle annotator would not add any new annotation.
2. None of the annotations made by the annotators were removed by the principle annotator if both of the annotators marked them earlier.
3. Annotations were added or removed if they were marked by either of the annotators provided that they both agree with the ‘principle annotator’ on the consensus decision complied thereafter.
4. In case of partially overlapping annotations, only the conflicting words were removed. For instance, the first independent annotator marks “might be involved” whereas the second annotator marks “might be”, then the decision will be made to resolve the annotation by removing the word “involved”.

The same workflow as mentioned above was used in annotating the “Remote corpus” mentioned later in the manuscript.

HYPO-TEST corpus serves as our gold standard for the performance evaluation of different hypothesis detection approaches (pattern matching and ML based) mentioned in the manuscript.

To address the concern of data bias that might result from our search and corpus assembly strategy, we also evaluated the performance of our model for speculation detection on Bioscope, an independent and expert-curated corpus. This corpus consists of medical and biological text annotated by experts for speculation and their linguistic scope. The corpus is a good resource for comparison and independent assessment of Natural Language Processing (NLP) systems [12]; hence, we used this corpus as a surrogate system to independently assess performance of our model (mentioned under result section).

**Figure 1:**
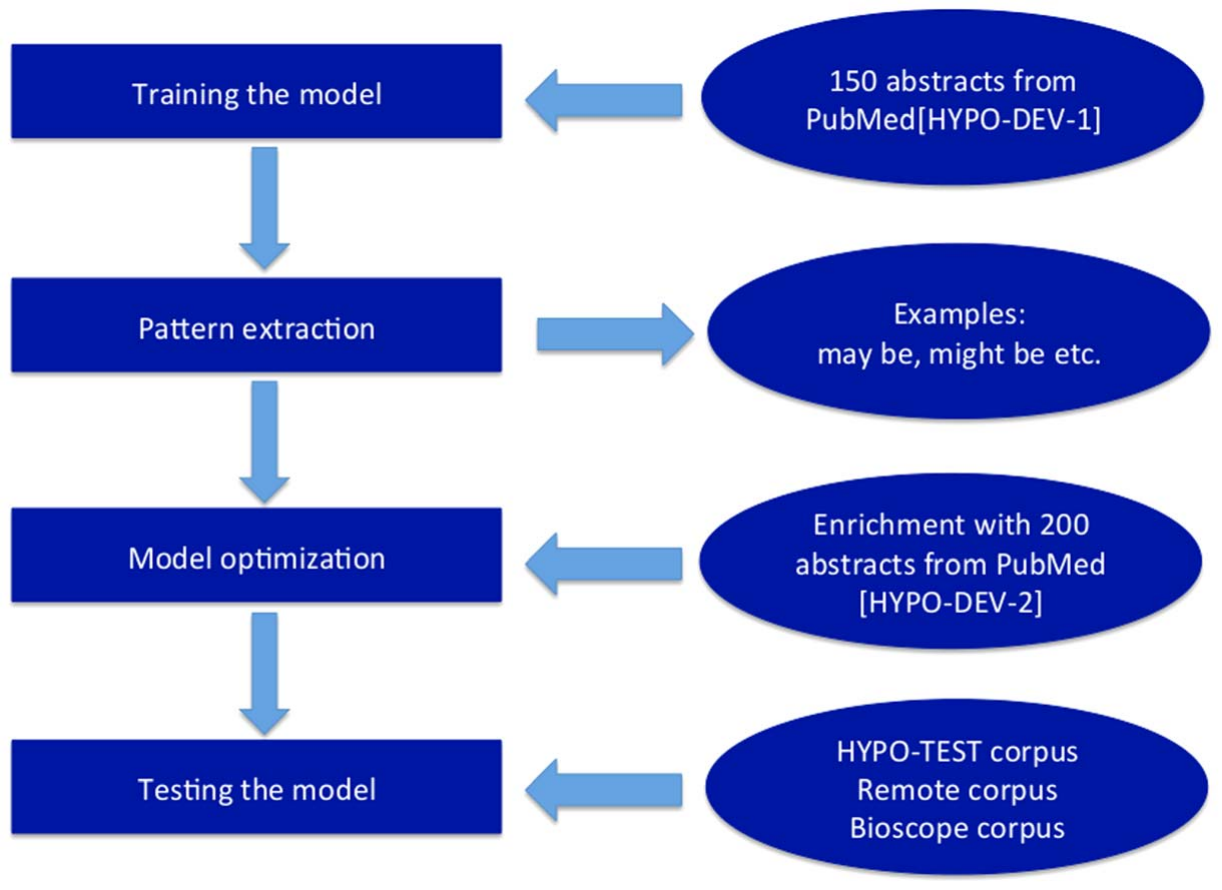
An overview of HypothesisFinder development approach. The workflow for the development of HypothesisFinder shows how the model was trained, optimized and on what data sets its performance was evaluated.

An overview of the methodology described in this section is provided in (Figure 1).

### Annotation of speculative patterns

Words and phrases such as ‘may be involved’, ‘might regulate’, ‘seems to play’, and ‘it appears as’ typically render statements as speculative, and are thus called speculation keywords or speculative patterns. These words are strong indicators of speculation in text. Particularly in scientific abstracts, whose purpose is to summarize the scientific knowledge presented in full text articles, these speculative patterns act as a linguistic marker that guides readers in detecting proposed hypotheses within text. In this work, annotations were performed in a way to capture not only speculative patterns (typically a phrase or a span of text) but also hypothetical sentences (sentences that also indicate a scientific hypothesis).

**Figure 2:**
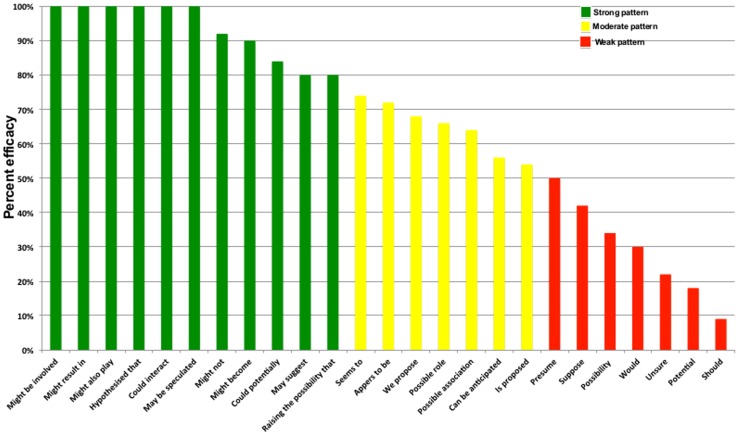
Classification of speculative patterns. Figure presents examples of strong, moderate and weak speculative patterns along with their estimated ‘percent efficacy’ or ability of pattern to cast a sentence as speculative.

Based on our experiences obtained from the annotation process, speculative patterns can be further classified as strong, moderate and weak patterns. Further to back up this claim, we parsed a new Alzheimer's related corpus of 58,922 abstracts into 707,946 sentences. Previously identified speculative patterns were searched for their presence in this ‘sentence corpus’ and a sentence count (i.e. total number of sentences with specific speculative pattern) was obtained for each pattern. Additionally, two independent curators manually curated the retrieved sentences (sentence count up to 50) for each specific pattern, hence calculating the ‘percent efficacy’ of each pattern to indicate a sentence as speculative. In most cases when the sentence count exceeded more then 50, we randomly chose 50 sentences for our analysis. An example of utilizing a strong, moderate, and weak pattern categorization is shown in (Figure 2).

Based on the results, we concluded that phrases containing modal verbs such as ‘may’, ’might’, or ‘could’ linguistically represent class of strong speculative identifier. Other words such as ‘potential’, ‘possibility’, ‘should ‘, ‘would’, and similar ones are not necessarily used in speculative context and may produce false positives. For instance, consider the following sentences:

1. *Herein, we discuss the evidence supporting the critical role of tau oligomers in AD, the* ***potential*** *and challenges for targeting them by immunotherapy as a novel approach for AD treatment..*(PMID: 21605039)
2. *For some forms like vascular dementias, the* ***possibility*** *of prevention is the most valuable approach that should be enforced more aggressively. (PMID: 17012946)*

To overcome this problem and to increase the accuracy of our model, we combined weak speculative patterns either with additional speculative words or with additional auxiliary verbs or adjectives before including them in the final dictionary. Revision of annotated patterns after introducing these changes to the dictionary showed improved performance and hence increased the quality of annotations as exemplified by following sentences:

1. *These data suggested that GHE* ***could be a potential*** *agent for preventing or retarding the development or progression of Alzheimer's disease.* (PMID:19221423)
2. *These results suggest that some SAD may involve alternative processing of multiple γ-secretase substrates,* ***raising the possibility that*** *the molecular pathogenesis of SAD might involve γ-secretase dysfunction.* (PMID:21681798)

In both of these cases, combining ‘potential’ and ‘possibility’ with additional speculative patterns such as ‘could be a’ and ‘raising the … that’ respectively turns these weak indicators to linguistically stronger identifiers of speculation, resulting in less likelihood of producing false annotations. Furthermore, to decrease false annotations and to better define what a speculative sentence is, annotation guidelines were also enriched with examples of patterns and sentences (Negative controls) that can conflict with speculative patterns and sentences.

The followings are some examples of sentences expressing inferences, results and conclusions, arguments and open questions, which cannot be considered speculative.

‘Inferences’

*Taken together these data demonstrate that this transgenic AD model* ***can serve*** *as a powerful tool for the identification of AD therapeutic interventions.* (PMID:21673973)

‘ Results and conclusions’

*Galantamine reduces behavioral symptoms in patients with mild to moderate AD, leading to reduced caregiver burden.* (PMID:21615354)

‘Argumentative sentences’

*There is increasing evidence that the consumption of flavonoid-rich foods* ***can beneficially*** *influence normal cognitive function.* (PMID:21982844)

‘Open questions’

*Whether T2D can cause late onset Alzheimer's disease (LOAD) remains to be elucidated.* (PMID:22433668)

Following the recommended annotation guidelines, abstracts in HYPO-DEV- 1, HYPO-DEV- 2, and HYPO-TEST datasets were annotated for speculative patterns and hypothetical sentences in text. Speculative patterns appearing in HYPO-DEV- 1 and HYPO-DEV- 2 were extracted to generate a dictionary. Synonymous patterns were manually detected and grouped together to represent constitutive patterns (see the next section for details). Synonymous patterns denote different permutations or combinations of a speculative pattern that can occur within text. For example, the representative speculation pattern ‘appear to’ could have the following synonymous patterns: *appeared to be|appears to be|appear that|appears that|appeared that |appearing to|appears to|appears to play|appear related|appeared related |appears related.*

### Dictionary characteristics and automatic pattern recognition

The dictionary used for recognition of speculative patterns in text comprises 156 representative and 392 synonymous patterns (Dataset S1). Efforts have been dedicated to cover all possible variations of speculative patterns appearing in developmental corpora. For automated identification of patterns within the HYPO-TEST corpus, ProMiner [13], a dictionary-based named entity recognition (NER) system, was used. In addition to dictionary-based NER, ProMiner also applies pre-defined rules and its search algorithm is geared towards handling the recognition of ambiguous multi-word terms in text. Previous scenarios are present where ProMiner has been used to perform NER by pattern matching or regular expressions in text [14]–[16]. The ProMiner search was performed using case-insensitive, word order-sensitive and the longest string exact match search constraints.

**Figure 3:**
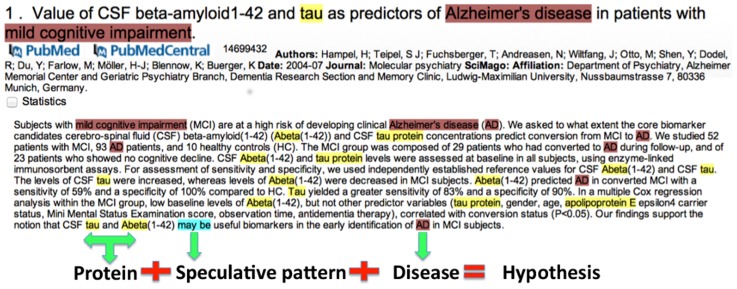
Example showing usage of HypothesisFinder integrated in SCAIView for extracting hypotheses related to Alzheimer's disease. Figure shows how HypothesisFinder is used within SCAIView in conjugation with other pre-indexed terminologies and ontologies to retrieve Alzheimer-specific hypotheses. Presented example shows how a hypothesis positioning Tau and Amyloid-beta as potential biomarker candidates in relation to AD is identified by HypothesisFinder in scientific abstracts.

To study real-use case scenarios, ProMiner along with the speculative pattern dictionary was applied to the complete MEDLINE abstracts for the identification of speculative patterns. The recognized patterns were indexed and visualized using SCAIView, a scalable indexing and retrieval platform that has exhibited successful information retrieval scenarios from MEDLINE [14], patents [15] and e-health records [16]. SCAIView supports document retrieval as well as entity extraction. Speculative statements are indexed and made searchable within SCAIView and can be searched in combination with other biomedical terminologies and ontologies. Thus, the hypothetical space related to a particular question of interest can easily be retrieved using SCAIView. An example of such search scenarios using SCAIView is shown in (Figure 3). Moreover, ‘HypothesisFinder’ has been integrated into SCAIView and is freely available for usage and testing at http://www.scaiview.com/scaiview-academia.html

### Training a machine learning model for hypothetical sentence detection

The application of ML-based approaches for sentence classification has demonstrated considerable success in the past [17]. To test whether dictionary-based or ML-based approach performs best for the identification of speculative statements in scientific text, we did a comparative assessment of an established ML-based approach against the pattern-based approach described above.

First, the HYPO-DEV-1 and HYPO-DEV-2 datasets were segmented into sentences. As required for ML training, sentences that represented hypotheses, i.e. contained speculative pattern annotations, were labeled as “POSITIVE” whereas those that did not were labeled as “NEGATIVE”. The overall training set contains 483 sentences labeled as POSITIVE and 2049 sentences labeled as NEGATIVE.

Sentences present in HYPO-TEST corpus formed an independent test set over which the performance of the trained model was validated. Similar to the training data, POSITIVE and NEGATIVE instances were generated for the test set, resulting in 246 sentences labeled as POSITIVE and 1194 sentences labeled as NEGATIVE.

A ML-based sentence classifier developed by Gurulingappa et al [16] was applied to train a sentence classification model (i.e. over HYPO-DEV-1 and HYPO-DEV-2 sentences). The sentence classifier facilitates switching between different classification algorithms that include Naïve Bayes (NB), Nearest Neighour (NN), Decision Trees (DT), Maximum Entropy (MaxEnt), and Support Vector Machines (SVM). The performance of sentence classification system was tested under three conditions using baseline features, speculative features, and lexico-syntactic features. Baseline features use all words appearing in sentences as features for classification. Speculative features were formed by occurrences of hand-crafted patterns (mentioned in Section *Annotation of speculative patterns*) that are potential indicators of hypotheses and Lexico-syntactic features were formed by the following:

- Lemmatized tokens appearing in sentences
- Bigrams of lemmatized tokens appearing in sentences,
- Trigrams of lemmatized tokens appearing in sentences,
- 2,3, 4 character prefixes and suffixes of nouns appearing in sentences, and
- Stanford parser generated token dependencies occurring in sentences.

During training and testing the model with lexico-syntactic features, the above-mentioned textual features were extracted from HYPO-DEV-1, HYPO-DEV-2, and HYPO-TEST sentences.

## Results

### Dictionary-based performance evaluation

The performance of HypothesisFinder was first evaluated on the HYPO-TEST corpus addressing the following two aspects:

1. The ability to recognize speculative patterns in text using a predefined pattern dictionary. The evaluation was performed by systematically comparing human annotated speculative patterns in the HYPO-TEST set with patterns identified by ProMiner.
2. The ability to classify sentences as ‘Hypothetical’ or ‘Non-hypothetical’ based on the recognition of speculative patterns. If ProMiner recognizes one or more speculative patterns in a sentence, the sentence is labeled as ‘POSITIVE’ where as sentences that do not contain speculative patterns are labeled as ‘NEGATIVE’. This classification also provides a rationale for the comparison of the pattern-based approach against the ML-based approach.

Evaluation metrics used for speculative pattern recognition and hypothetical sentence classification were Precision, Recall and F-score. The following formulas were used for the computation of Precision, Recall and F-score values [18].
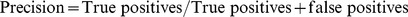


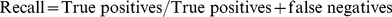


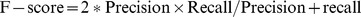
where true positives are the number of entities/sentences that were annotated by ProMiner and further matches with the human annotated entities/sentences that serves here as our gold standard. False positives are the number of entities/sentences that were recognized by ProMiner, but were not matched to annotations in the gold standard. False negatives are the number of entities/sentences that were not found by ProMiner when compared with the gold standard annotations. The results of the evaluation are listed in (Table 1).

**Table 1:** Performance of HypothesisFinder on the HYPO–TEST corpora.

Method	Tool	Precision	Recall	F-score
Pattern identification	ProMiner	0.84	0.86	0.85
Sentence classification	ProMiner	0.91	0.95	0.92
Sentence classification	MaxEnt classifier (*lex features*)	0.90	0.47	0.62
Sentence classification	MaxEnt classifier (*base features*)	0.85	0.35	0.50
Sentence classification	MaxEnt classifier (*lex+base features*)	0.83	0.46	0.59
Sentence classification	MaxEnt classifier (*lex+spec features*)	0.88	0.89	0.88
Sentence classification	MaxEnt classifier (*base+spec features*)	0.83	0.88	0.85
Sentence classification	MaxEnt classifier (*base+spec+lex features*)	0.84	0.88	0.86

MaxEnt indicates Maximum Entropy classifier. Applied features sets were baseline features (*base*), speculative features (*spec*), lexico-syntactic features (*lex*), and their combinations.

For ‘Pattern recognition’, HypothesisFinder achieved a precision of 0.84 and a recall of 0.86 in comparison to the manual annotation in the HYPO-TEST corpus. The major reason accountable for a 14% loss in coverage seems to be deletion of very weak speculative patterns in their original form to avoid chances of false annotations, as shown with the examples in “Annotation of speculative patterns” section.

### Performance evaluation of sentence classification by machine learning

The performance of the maximum-entropy based sentence classification model was evaluated on a corpus composed of HYPO-TEST sentences. Precision, Recall and F-score metrics used for sentence classification over the class ‘POSITIVE’. Initially, experiments were performed by switching between different classification algorithms provided within the sentence classification system. Among the different ML classifiers tested, the Maximum entropy classifier (MaxEnt) provided the best performance whose results are considered here.

Comparison of the performance of ProMiner and MaxEnt classifier in their ability to classify sentences in HYPO-TEST as hypothetical is shown in Table 1. The MaxEnt classifier was applied for sentence classification using baseline features, speculative features, lexicosyntactic features, and their combinations. The MaxEnt classifier achieved the F-score of 0.50 and 0.62 when using simple words or lexicosyntactic features, respectively. Adding the speculative features boosted the ML classification performance with the F-score of 0.88. This indicates the value of hand-derived speculative patterns in assisting the development of a robust machine-learning model. Classification using speculative features alone was not possible since these features do not appear in all sentences whereas the ProMiner-based classification resulted in the F-score of 0.92.

The sentence classifier used here was applied with default input parameters (as defined by Gurulingappa et al). Since the goal of this work was to evaluate the adaptability of pattern-based approach to hypothetical statement detection, no extensive optimization of features for the sentence classifier was performed. Nevertheless, the observable difference in performance of pattern-based approach as compared to ML-based approach drives the interest in applying ‘easy-to-mold’ patterns for identifying hypothetical statements particularly in scientific abstracts where scientific knowledge presented in articles is summarized.

### Performance assessments over Bioscope and Remote corpus

Evaluation of our model on Bioscope corpus (Table 2) in comparison to HYPO-TEST corpus show a relatively lower recall (0.73) while a comparably high precision (0.91) is maintained. A possible reason for the recall decrease is the lack of “weak patterns” (i.e. general terms such as ‘should’, ‘would’, ‘potential’) in HypothesisFinder's dictionary; these weak patterns are marked as speculative within the Bioscope corpus. Such weak patterns were integrated into the dictionary only in combination with other terms (e.g. ‘would likely’, ‘should possibly’, ‘could be potential’) so that their viability for identifying speculative expressions compared to their basic form is improved. Final assessment of our model on Bioscope corpus indicated a good performance with the F-score of 0.81, further confirming the ability of our methodology to detect speculations in text without corpus bias.

**Table 2:** Performance of HypothesisFinder over Bioscope and Remote corpus.

Data type	Source	Precision	Recall	F-score
Bioscope (abstracts)	Bioscope corpus	0.91	0.73	0.81
143 abstracts related to AD	AlzSWAN/PubMed	0.90	0.97	0.93
100 abstracts related to Epilepsy	PubMed	0.92	0.88	0.89
100 abstracts related to Parkinson's disease	PubMed	0.86	0.89	0.87

Performance of HypothesisFinder was also checked on a so-called ‘Remote corpus’ comprising AlzSWAN, Parkinson and Epilepsy corpus. AlzSWAN corpus comprises 143 abstracts presented as primary reference of hypothetical statements quoted in the AlzSWAN knowledge base. Again for calculation of evaluation metrics, each of the sub-corpora was manually annotated for speculative statements based on the previously defined annotation guidelines (mentioned under section corpus characteristics and annotations). The results of this evaluation, listed in Table 2, show that HypothesisFinder was able to detect speculative statements with 90% accuracy and 97% sensitivity in AlzSWAN corpus.

In order to show domain-independent application of HypothesisFinder, we also tested our model for speculation detection on a sub-corpus of randomly derived abstracts from PubMed related to Epilepsy and Parkinson's disease. Again, the high accuracy and sensitivity performance of HypothesisFinder (Table 2) demonstrates that our approach can be extrapolated to any given topic of interest.

### Application of HypothesisFinder: A case study

Since our initial motivation for developing ‘HypothesisFinder’ was to establish an automated approach for capturing the breadth of hypotheses existing in the scientific literature around AD, we tested and benchmarked our automated approach against the expert-curated AD-specific hypothetical knowledge (inferred from the literature-derived speculations) in the AlzSWAN database. For this purpose, hypothetical statements were extracted automatically using HypothesisFinder and the results were compared to the AlzSWAN hypothesis browser. We sought to explore the power of HypothesisFinder in a semantically enhanced environment such as SCAIView by including a human gene dictionary and the Alzheimer's disease ontology [19] in the search. Through the combination of these dictionaries, a link between hypothetical statements and molecular (e.g. proteins) and clinical (e.g. disease stage) features of Alzheimer's disease can be established.

**Figure 4:**
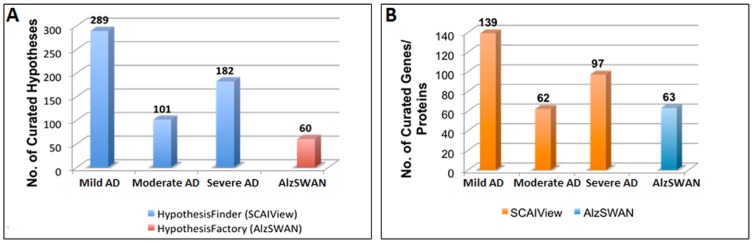
Comparison of information densities: HypothesisFinder vs. **AlzSWAN.** A- The statistical comparison between the numbers of hypotheses related to AD captured by HypothesisFinder within SCAIView (s*tage-specific retrieval*) and the hypotheses with extended annotation derived from citations mentioned in the AlzSWAN database. B- A comparison between biological entity retrieval using SCAIView and relevant entries in AlzSWAN.

Figure 4 (A) illustrates the comparison between Alzheimer's stage-specific hypotheses linked to their corresponding biological entity (i.e. genes or proteins) extracted by HypothesisFinder as integrated in SCAIView (See Dataset S2, S3, and S4) and hypotheses with extended annotations (i.e. linked to genes and proteins) present in the AlzSWAN database. It is evident from our analysis that HypothesisFinder collects a large number of speculative statements, significantly more than the number of hypotheses listed in AlzSWAN. Even after curation of the hypotheses identified by HypothesisFinder, the count of normalized genes and proteins linked to these hypotheses (Figure 4 (B)) is higher than those in the AlzSWAN database. Additionally, we were also able to link extracted hypotheses to clinically established AD stages. In order to analyze the coverage of relevant genes and proteins linked to the hypotheses, we also compared the lists of genes and proteins mentioned in AlzSWAN with the list of entities retrieved by SCAIView. The comparison showed that all genes or proteins represented in AlzSWAN are actually a subset of the list generated by the HypothesisFinder approach, which indicates that HypothesisFinder is extending the hypothetical knowledge space not only at the level of speculative statements but also at the level of molecular entities being linked to disease hypotheses. Nonetheless, it should be noted that our automated workflow is not a replacement for expert-curated knowledge inference such as the one used by AlzSWAN but rather it is complementary to extensive manual curation efforts by domain experts. Therefore, systematic assembly of domain specific speculative knowledge present in Medline as well as full text documents using automated approaches will improve the knowledge aggregation and discovery process, leading to an accelerated growth of knowledge bases such as AlzSWAN. However, for making inferences over the gathered hypotheses, human intervention and expert curation is mandatory, as it is done in AlzSWAN.

### Chronological analysis of hypothesis-related knowledge on AD

**Figure 5:**
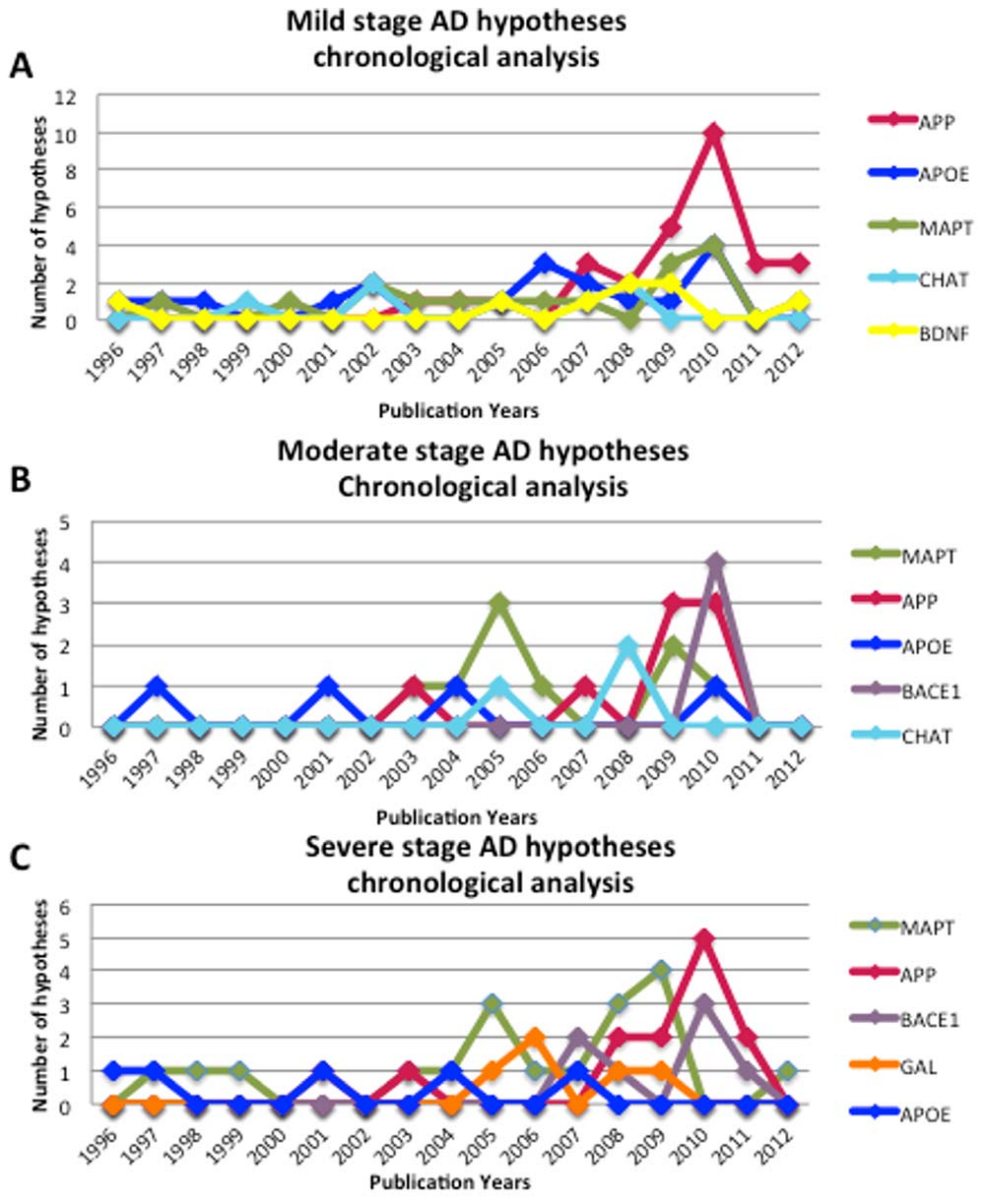
Chronological order of hypotheses proposed in Mild (A), Moderate (B) and Severe (C) AD. Figure shows a schematic representation of how AD stage specific hypothesis related to top five genes that are high-frequently investigated in the literature has evolved in number over time Abbreviations mentioned stands for Amyloid beta (A4) precursor protein (APP), Apolipoprotein E (APOE), Microtubule- associated protein tau (MAPT), Choline O-acetyltransferase (CHAT), Brain-derived neurotrophic factor (BDNF), Beta-site APP-cleaving enzyme 1(BACE 1), Galanin prepropedtide (GAL).

New scientific findings and discoveries that are expressed in the form of speculative statements represent the category of so-called ‘transient articles,’ whose citations peak within a short period of time [20]. These articles usually contain emerging trends of a specific knowledge domain, which evolves over time. To show how the intellectual landscape of scientific hypotheses on AD has evolved, we mapped the chronological order of retrieved hypotheses linked to five genes that are frequently speculated in the literature to be involved in the disease. As is shown in (Figure 5), the AD knowledge domain is currently dominated by speculations around APP (Amyloid beta (A4) precursor protein) and MAPT (Microtubule- associated protein tau) across all disease stages. Moreover, it is evident that the number of proposed hypotheses across all stages has been increasing since 2005.

### Construction of hypothetical stage-specific disease networks for AD

**Figure 6:**
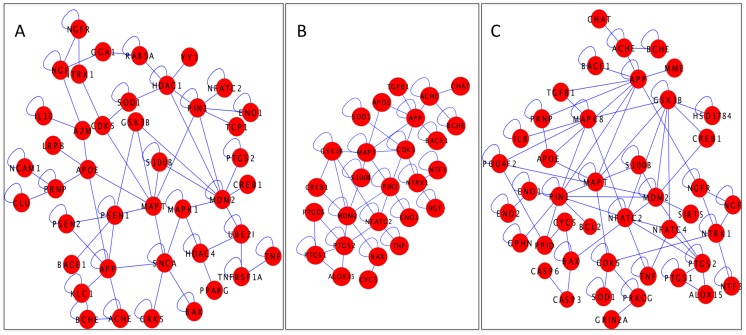
Stage specific AD networks. Figure presents protein interaction networks for Mild(A), Moderate(B), Severe(C) stage of Alzheimer's disease. These stage specific networks have been generated by using BioNetBuilder plugin in Cytoscape, which was given genes and proteins, associated to stage-wise hypotheses as input.

In order to show the application value of HypothesisFinder in disease modeling, we used the BioNetBuilder plugin [21] in Cytoscape to build hypothetical protein interaction networks from the set of genes and proteins linked to disease stage-specific hypotheses. (Figure 6) depicts the connected components of three networks representing the three stages in AD. Such stage-specific disease networks visualize the disease progression at the molecular level and can provide a framework for further integrative analyses; e.g. integration of stage-specific gene expression data into network models could help to analyze gene expression perturbations across the three stages of AD.

## Discussion

Hypothesis-encoding statements comprise a good part of a researcher's expectation about the relationship between two or more biological variables [22]. Discovery of new rules and causal relationships linking genotype to phenotype remains one of the main challenges in life sciences [23]. Accordingly, linking hypotheses to the established knowledge or background theory can strengthen the ability of hypothesis-driven data integration across different levels of biological systems.

In order to automatically extract hypothetical information from the literature and link the genotypic features (i.e. genes and proteins) to the phenotypic features of AD (i.e. disease stages), we started with two independent technological approaches (pattern and machine learning based) towards solving the problem of identifying speculative statements in scientific text. A comparative evaluation of both approaches showed that our pattern-based approach outperforms the machine learning-based approach in specific scenario since speculation is represented in text with the help of certain enumerable patterns. As shown in Figure 2, the pattern matching approach can substantially improve the recognition of speculative statements particularly in scientific abstracts where authors present the core of their research in limited paragraphs. Hence, a speculative pattern, if found, ascertain a presented hypothesis. Nonetheless, authors do believe that there is a room for development of dedicated machine learning modules for hypothesis detection. However, the goal of the presented work was to evaluate the adaptability of pattern-based approach to hypothetical statement detection and not extensive optimization of features. Research into feature selection, instance selection, sequence-based learning, and the integration of pattern and machine-learning approaches will be thoroughly conducted in follow-up studies.

The acceptable performance of our pattern-based approach in extracting hypothesis-encoding statements from free text indicates that automated information extraction could possibly reduce human reading and curation efforts for enrichment of knowledge bases, provided that the performance of such technology is comparable to the quality of human expert curation. Another advantage of automated harvesting of scientific hypotheses and speculative statements is that dedicated searches can be aimed at capturing the complete spectrum of speculative statements within a domain.

As shown in the case of complex, mostly idiopathic diseases like AD, by formulating a series of reasonable speculations on causes and effects, we gained interesting insights into the disease's staging and progression at the molecular level. Each of the extracted AD hypotheses posits a specific relationship between involved genes/proteins and their corresponding disease phenotype. Such relationships can guide researchers in developing new experiments to test the proposition. Exploration of these hypotheses provides an overview on emerging knowledge niches, which have the potential to add value to ongoing research activities. Speculative patterns linked to molecular entities, when expressed in the abstracts, represent relevant hypothetical knowledge that can be systematically collected and used for modeling purposes as demonstrated above. The patterns used in this work to detect speculative statements in text are of a general nature, which extends the scope of their applicability beyond the AD domain, as shown in the Epilepsy and Parkinson's disease scenarios.

We believe that the method developed in the course of this work will prove very useful for biomedical research. HypothesisFinder allows for the systematic collation and analysis of reported speculative findings in a specific context. This can have a tremendous consequence for health-related studies; for instance, it could be used to understand initial speculated mechanisms or modes of action that led to the success or failure of drugs. Systematic collection of hypotheses allows for rationalization of discussions about possible interpretations of data. Since speculations represent the gray zone of scientific knowledge, they can provide incremental support to the main hypothesis underlying the research. Conversely, if the speculations are contradictory then they could shift the direction of the research towards new and rewarding avenues. Captured hypothetical knowledge can be used to model diverse disease scenarios. Our use of this technology on the etiology of AD is driven by our interest in modeling neurodegenerative diseases at the systems level. In the future, we intend to build a service around HypothesisFinder that can systematically identify and rank new emerging hypotheses in different disease areas. We will also conduct additional research into distinguishing novel hypotheses from those already existing, and we will use this capability to set up automated alerts for notification of novel hypotheses.

## Supporting Information

Dataset S1 Dictionary of various speculative patterns and their corresponding synonyms.
(TXT)

Dataset S2 Hypothesis related to Mild stage of AD extracted by using HypothesisFinder in combination with Alzheimer's disease ontology (ADO) and Human gene and protein dictionary within SCAIView.
(XLS)

Dataset S3 Hypothesis related to Moderate stage of AD extracted by using HypothesisFinder in combination with Alzheimer's disease ontology (ADO) and Human gene and protein dictionary within SCAIView.
(XLS)

Dataset S4 Hypothesis related to Severe stage of AD extracted by using HypothesisFinder in combination with Alzheimer's disease ontology (ADO) and Human gene and protein dictionary within SCAIView.
(XLS)
